# Effects of Ethanol Treatment on Storage Quality and Antioxidant System of Postharvest Papaya

**DOI:** 10.3389/fpls.2022.856499

**Published:** 2022-06-14

**Authors:** Zhichao Liu, Fan Jiang, Yiming Mo, Haida Liao, Ping Chen, Hongna Zhang

**Affiliations:** ^1^Sanya Nanfan Research Institute of Hainan University, Hainan Yazhou Bay Seed Laboratory, Sanya, China; ^2^Key Laboratory for Quality Regulation of Tropical Horticultural Crops of Hainan Province, College of Horticulture, Hainan University, Haikou, China

**Keywords:** papaya, ethanol, postharvest physiology, storage quality, antioxidant system

## Abstract

Papaya is the fourth most favored tropical fruit in the global market; it has rich nutrition and can be used for medicine and food processing. However, it will soften and mature in a short time after harvest, resulting in a lot of economic losses. In this study, papaya fruits were soaked in 0, 12.5, 25, 50, and 100 ml/L ethanol solutions for 2 h and stored at 25°C for 14 days, by which we explored the effects of ethanol treatment in papaya after harvest. At an optimal concentration of ethanol treatment, color changing of the papaya fruits was delayed for 6 days, and decay incidence and average firmness of the fruits were shown as 20% and 27.7 N, respectively. Moreover, the effect of ethanol treatment on antioxidant systems in the papaya fruits was explored. It was observed that ethanol treatment contributed to diminish the development of malondialdehyde (MDA), ethylene, and superoxide anions. Furthermore, the activities of superoxide dismutase (SOD), catalase (CAT), and ascorbate peroxidase (APX) were promoted than those of control group, while the activities of peroxidase (POD), phenylalanine ammonia-lyase (PAL), and polyphenol oxidase (PPO) were brought down. In addition, the principal component analysis (PCA) showed that PAL, ethylene, and superoxide anions were the main contributors for the maturity and senescence of postharvest papaya. In this experiment, ethanol treatment had the potential of delaying the ripening and maintaining the storage quality of papaya fruits.

## Introduction

Papaya (*Carica papaya* L.) is a rapid-growing, latex-producing herbaceous fruit tree and is widely cultivated in the tropics and subtropics (Rogayah et al., 2018). As the fourth most favored tropical fruit in the global market after bananas, mangoes, and pineapples (Xu et al., 2020), the papaya fruit has rich nutrients, carotenoids, and vitamins A and C, as well as minerals such as Mg, K, and Cu (Hardisson et al., 2001; Wall, 2006). In addition to fresh-eating, papaya fruit can also be used for medicine (Karunamoorthi et al., 2014) and food processing (Mendoza et al., 2008), and contains unique papain for meat tenderization and beer clarification (Liu et al., 2007). Its seed oil can be extracted as a green biofuel (Asokan et al., 2018).

Papaya is a typical respiratory climacteric fruit, which will soften and mature in a short time after harvest. During ripening, the yellowing of the outer pericarp begins at the tip of the stigma, while coloring and softening of the inner flesh develops outward from the endocarp (Paull et al., 1997). During maturation, soluble solids content of papaya towards increase, which varies from 6% to 19% depending on varieties (Sivakumar and Wall, 2013). The ascorbic acidity of “Golden” papaya increases by 20–30% during maturation, while the pericarp h° value and titratable acid content decrease (Bron and Jacomino, 2006). The total carotenoid content of “Pococi” papaya improves from 130 μg/100 g FW to 5,414–6,214 μg during maturation, and the esterified β-cryptoxanthin content is very high at the early stage of maturation. At the later stage, β-cryptoxanthin ester and (all-E)-lycopene are dominant (Schweiggert et al., 2011). A study has shown that the maturation of the papaya fruit is associated with increased activities of polygalacturonase (PG), pectate lyase (PEL), catalase (CAT), and ascorbate peroxidase (APX) and high level of H_2_O_2_ and lipid peroxidation, as well as decreased activities of superoxide dismutase (SOD) and guaiacol peroxidase (GPX) (Pandey et al., 2013).

Ethanol (C_2_H_5_OH), a low-toxic plant secondary metabolite, is used as an effective and safe food additive for postharvest preservation and storage of horticultural crops. It was found that ethanol soaking or steam treatment could prolong the life of freshly cut Bougainvillea flowers (Hossain et al., 2007) and inhibit the maturation of melons (Liu et al., 2012) and rottenness of “Red Earth” grapes (Candir et al., 2012). Ethanol vapor treatment repressed chlorophyll catabolic enzyme and related gene expression, which postponed the yellowing of broccoli (Fukasawa et al., 2010). Besides, ethanol treatment could retard the senility of fruits by killing bacterial propagules, fungi, and viruses. Treating bayberry with 1 or 1.5 mL/L ethanol for 2–3 h and 0.5 mL/L ethanol for 3 h could significantly inhibit the germination of pathogenic bacteria and delay the aging of fruits (Wang et al., 2011).

In this study, physiological changes in papaya were studied using different concentrations of ethanol immersion as treatment, aiming to explore the optimal concentration of ethanol treatment for papaya postharvest storage, enrich the theory of papaya postharvest preservation, and provide some reference for production practice.

## Materials and Methods

### Materials and Treatments

Papaya fruits (*C. papaya* L., cv ‘Sunrise’), harvested in Ledong county, Hainan province, were quickly transported to the laboratory for treatments. A total of 150 papaya fruits with similar size and maturity degree of 70–80% and without mechanical damages, diseases, and insect pests were randomly divided into five groups, soaked in 0.05% sporgon solution for 5 min, and dried naturally. Then, the fruits were soaked in 0, 12.5, 25, 50, and 100 mL/L ethanol solutions for 2 h (which were separately regarded as 0 mL/L ethanol treatment (CK) for the control group and treatment groups A, B, C, and D). After drying the surface of the pericarp, the fruits were transferred to the storage place at 25°C and 80% RH. Three fruits were selected randomly from each treatment group on days 0, 2, 4, 6, 8, 10, 12, and 14 (the day of ethanol treatment was regarded as day 0) for determination of physiological indicators, cryopreserved with liquid nitrogen, and preserved in a refrigerator at −80°C for further analysis.

### Measurement of Physiological Indicators

#### Determination of Decay Incidence and Weight Loss Rate

Ten fruits from each treatment group were selected for observation of decay incidence. When there appeared rottenness, mildew, or black-brown spots on a fruit, it was regarded as decay and recorded.

Weight loss rate was calculated by weighing the fruits on an electronic balance. The formula is as follows:


$${\mathrm{WLR}}_{n}=\frac{w_{0}-w_{n}}{w_{n}}\times 100\%,$$

where WLRn represents the weight loss rate of the fruit on day N, w_0_ means the weight on the initiation day, and w_n_ means the weight on day N.

#### Determination of Firmness, Soluble Solid Content, and Titratable Acidity

Firmness was measured with a sclerometer. Points were chosen from the equatorial surface of each side of every fruit to measure firmness. Soluble solid content was determined with a handheld digital display glycosimeter (PAL-1; Atago Ltd., Japan). To determine titratable acidity, 5 mL papaya fruit juice was extracted, dissolved in 50 mL distilled water, added with phenolphthalein, and then titrated with 0.1 mol/L, pH 8.3 NaOH solution, and acidity was expressed as the equivalent percentage of malic acid.

#### Determination of MDA Content

The measuring method of MDA was based on Ding et al. (2015) and slightly modified. Pulp 2 g and 3 mL 0.05 M, pH 7.8 sodium phosphate buffer were homogenized in ice bath and then centrifuged at 4°C and 12,000 *g* for 15 min. A 2 mL 5 g/L solution of thiobarbituric acid was added into 2 mL supernatant, water bathed for 15 min at 95°C, cooled, and centrifuged for another 15 min. We determined OD_450_, OD_532_, and OD_600_ values and calculated MDA content using the formula:


$$C=6.452\times \left({\mathrm{OD}}_{532}-{\mathrm{OD}}_{600}\right)-0.559\times {\mathrm{OD}}_{450}.$$

#### Determination of Superoxide Anions Content

The determination method for superoxide anions content was referred to Cao et al. (2007). Pulp 2 g and 3 mL 0.05 M, pH 7.8 sodium phosphate buffer were homogenized in ice bath and then centrifuged at 4°C and 12,000 r/min for 20 min. We took 1 mL of the supernatant and added 1 mL of the sodium phosphate buffer and 1 mL of 1 mmol/L hydroxylamine hydrochloride solution. The solution was stirred well and kept at 37°C for 20 min. Then, 1 mL 17 mmol/L *p*-aminobenzene sulfonic acid solution and 1 mL 7 mmol/L α-naphthylamine solution were added, and the mixture was stirred and kept at 37°C for 20 min. OD_530_ value was measured, and the blank was adjusted with distilled water.

#### Determination of Ethylene Content

Ethylene content was determined using an enzyme-linked immunoassay (ELISA) kit (Jiangsu Kete, Jiangsu, China) and referred to Sun et al. (2016). We added papaya pulp samples and standard and antibodies marked with horseradish peroxidase (HRP) to microwells precoated with ethylene antibody and incubated the mixture at 37°C for 60 min. Then, 3,3′,5,5′-tetramethylbenzidine (TMB) was utilized for development of color, which turned blue under the action of HRP and finally became yellow under the activity of acid. Color depth had a positive correlation with ethylene content in the samples. We determined the absorbance at 450 nm and calculated the ethylene content of the samples.

#### Determination of Enzyme Activities and Total Protein Content

The activities of SOD, peroxidase (POD), CAT, phenylalanine ammonia-lyase (PAL), polyphenol oxidase (PPO), and APX, and total protein content were determined using assay kits (Nanjing Jiancheng, Jiangsu, China) and following the manufacturer’s specification.

### Data Processing and Analysis

This study utilized a completely randomized design with a total of three biological repeats. Data were analyzed by one-way analysis of variance (ANOVA). Means were compared using least significant difference (LSD) with a confidence level of *P* < 0.05. All statistical analyses were carried out with IBM SPSS Statistics (Version 26.0, IBM Corporation, United States).

## Results and Analysis

### Color-Changing of Postharvest Papaya Treated With Ethanol

**FIGURE 1:**
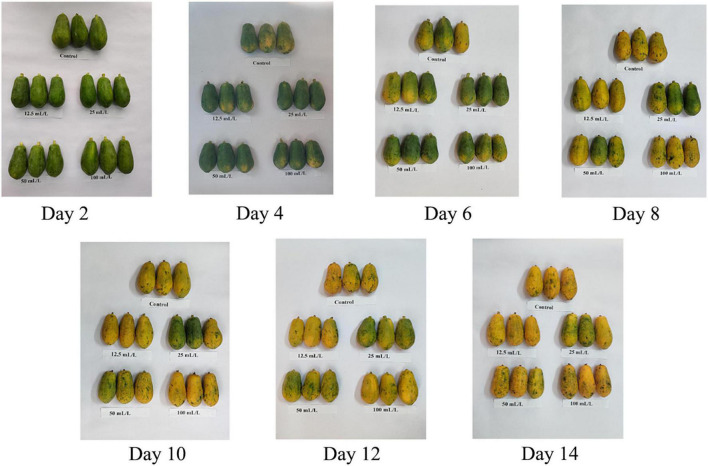
Color-changing of papaya fruits treated with 0, 12.5, 25, 50, and 100 mL/L ethanol within 14 days.

It can be observed from Figure 1 that on day 6 of storage, the papaya fruits of the control group began to change color, and that the surface turned yellow in a large area on day 8. For comparison, ethanol treatment had the effect of delaying color transformation, and the effect of treatment on group B (25 mL/L ethanol) was the most obvious, because the color was still green on day 12, followed by treatment group C (50 mL/L ethanol), in which the fruits partially turned yellow. The delayed effect on treatment groups A and D however, was not evident. It indicated that proper concentration of ethanol treatment could delay the color-changing of papaya fruits for 6 days.

### Appearance and Flavor Quality of Postharvest Papaya Treated With Ethanol

**FIGURE 2:**
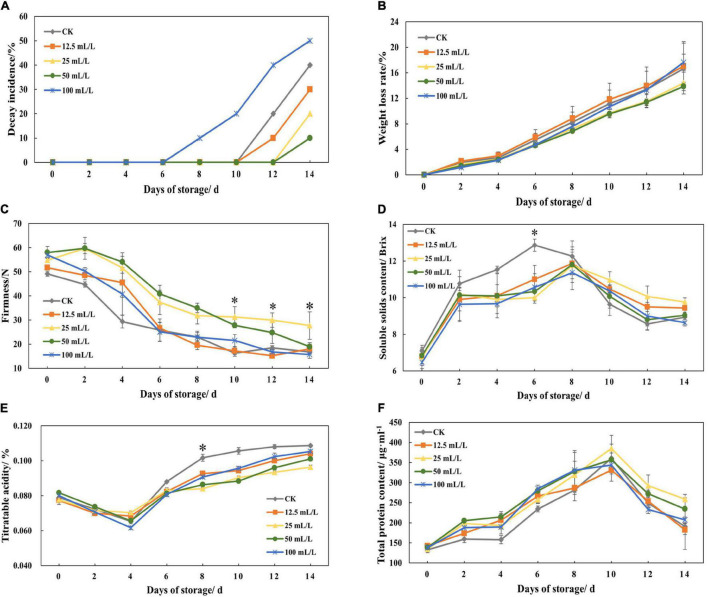
**(A)** Decay incidence, **(B)** weight loss rate, **(C)** firmness, **(D)** soluble solids content, **(E)** titratable acidity, and **(F)** total protein content of papaya fruits treated with 0, 12.5, 25, 50, and 100 mL/L ethanol. Values are means ± SD. *Represents significant differences among the treatments (*p* < 0.05).

After 14 days of storage, it can be observed that the decay incidence of treatment groups B and C decreased by 20 and 30% compared with the control group, and that weight loss rate decreased by 2.25 and 2.8%, respectively, while average fruit firmness increased by 65.37 and 13.13% (Figures 2A–C). In addition, low concentration of ethanol treatment is beneficial to storage, while higher concentration (treatment group D, 100 mL/L ethanol) was not conducive to storage and may even accelerate decay.

Soluble solid content and titratable acidity could be reduced by ethanol treatment. According to Figure 2D, the peak value of soluble solids content in the control group appeared on day 6 and then declined rapidly, while treatment groups reached peak value on day 8 and after that declined slowly. The titratable acidity of all the experimental groups reached troughs on day 4, but it increased rapidly in the control group and slowly in the treatment group in the following days (Figure 2E).

During storage, protein content generally increased slowly and peaked on day 10 (Figure 2F), which was because of the synthesis of a great number of enzymes in the period of respiratory climacteric. However, ethanol treatment had little effect on total protein content. At the later stage of storage, the total protein content of treatment group B was the highest, although the effect was not obvious.

### Contents of Malondialdehyde, Ethylene, and Superoxide Anions

**FIGURE 3:**
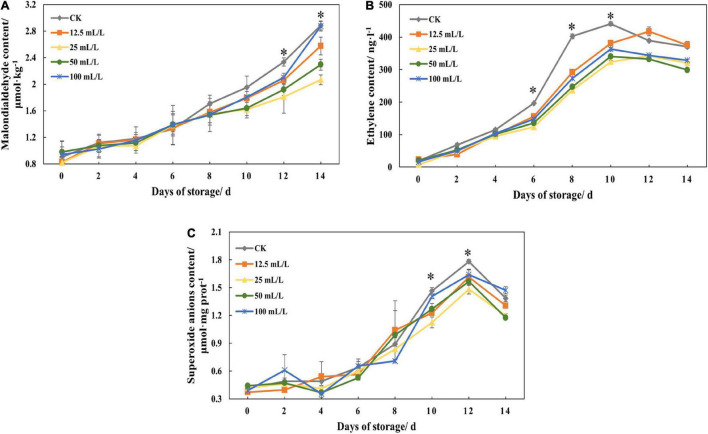
**(A)** Malondialdehyde content, **(B)** ethylene content, and **(C)** superoxide anions content of papaya fruits treated with 0, 12.5, 25, 50, and 100 mL/L ethanol. Values are means ± SD. *Represents significant differences among the treatments (*p* < 0.05).

Ethanol treatment could significantly reduce the content of MDA at the later stage of storage, and treatment group B had the best effect, followed by treatment groups C and A(Figure 3A). On day 12, the content of MDA in treatment groups A, B, and C was reduced by 11.78, 22.51, and 17.86%, respectively, compared with the control group (Table 1). In addition, there was no marked quantitative difference in MDA content between treatment group D and the control group on day 14, indicating that high concentration of ethanol treatment was unsuitable for storage.

**TABLE 1:** Effect of treatment on postharvest papaya after 8, 10, and 12 days of storage.

Treatments (days)		MDA	Ethylene	Superoxiade anions	SOD	POD	CAT	PAL	PPO	APX
		**μmol/kg**	**ng/L**	**μmol/mg prot**	**U/mg prot**	**U/mg prot**	**U/mg prot**	**U/mg prot**	**U/mg prot**	**U/mg prot**
8	Control	1.71 ± 0.13a	402.49 ± 7.55a	0.89 ± 0.07a	53.40 ± 0.95abc	299.43 ± 23.12a	11.17 ± 0.70b	7.44 ± 0.34a	22.44 ± 2.89a	0.16 ± 0.02a
	A	1.58 ± 0.16a	291.86 ± 4.83b	1.04 ± 0.32a	55.53 ± 1.12a	232.56 ± 5.54bc	12.19 ± 0.30b	5.88 ± 0.30b	16.33 ± 2.09a	0.16 ± 0.06a
	B	1.55 ± 0.13a	236.62 ± 3.82d	0.84 ± 0.03a	54.21 ± 0.24ab	209.29 ± 12.18c	14.26 ± 0.16a	3.38 ± 0.50d	13.09 ± 0.67a	0.27 ± 0.06a
	C	1.54 ± 0.25a	246.94 ± 1.83d	0.99 ± 0.26a	50.06 ± 1.04c	260.40 ± 1.98b	13.94 ± 0.15a	4.62 ± 0.25c	19.31 ± 5.96a	0.23 ± 0.07a
	D	1.54 ± 0.16a	273.13 ± 4.57c	0.71 ± 0.02a	51.68 ± 1.63bc	268.39 ± 2.58ab	13.45 ± 0.30a	6.82 ± 0.24ab	20.44 ± 2.78a	0.25 ± 0.04a
10	Control	1.95 ± 0.17a	440.91 ± 6.05a	1.46 ± 0.04a	47.04 ± 0.94c	258.10 ± 8.94c	12.47 ± 0.39b	10.12 ± 0.19a	36.72 ± 0.89a	0.19 ± 0.04c
	A	1.79 ± 0.15a	380.75 ± 4.81d	1.23 ± 0.03cd	52.68 ± 3.50bc	303.59 ± 11.62a	12.82 ± 0.34ab	8.18 ± 0.87bc	30.41 ± 0.63ab	0.21 ± 0.05c
	B	1.62 ± 0.13a	323.92 ± 4.73e	1.12 ± 0.06d	61.55 ± 0.99a	275.09 ± 3.44bc	12.64 ± 0.12ab	5.89 ± 0.14d	26.35 ± 1.90b	0.46 ± 0.02a
	C	1.64 ± 0.12a	340.43 ± 3.44d	1.27 ± 0.06cd	56.83 ± 1.42ab	291.12 ± 1.78ab	13.43 ± 0.31a	7.04 ± 0.59cd	33.46 ± 4.74ab	0.37 ± 0.02ab
	D	1.81 ± 0.08a	362.97 ± 5.19c	1.41 ± 0.02ab	48.30 ± 0.79c	311.31 ± 2.92a	12.24 ± 0.23b	9.53 ± 0.56ab	31.11 ± 4.46ab	0.28 ± 0.03bc
12	Control	2.34 ± 0.06a	389.16 ± 4.28b	1.78 ± 0.02a	30.28 ± 0.86d	224.52 ± 1.13a	10.03 ± 0.03a	7.37 ± 1.48a	23.00 ± 1.27a	0.22 ± 0.06b
	A	2.06 ± 0.07ab	417.41 ± 13.53a	1.63 ± 0.08ab	32.13 ± 1.00cd	217.39 ± 6.13ab	10.33 ± 0.22a	4.62 ± 1.21ab	18.59 ± 3.61a	0.30 ± 0.07b
	B	1.81 ± 0.24b	342.02 ± 4.84c	1.48 ± 0.05b	51.58 ± 2.65a	184.32 ± 2.88cd	10.65 ± 0.24a	3.62 ± 1.68b	17.16 ± 0.31a	0.49 ± 0.04a
	C	1.92 ± 0.09b	332.17 ± 4.73c	1.57 ± 0.04b	36.49 ± 0.78c	164.65 ± 9.00d	10.60 ± 0.53a	5.14 ± 0.61ab	18.98 ± 3.95a	0.42 ± 0.02a
	D	2.10 ± 0.06ab	343.92 ± 3.81c	1.64 ± 0.06ab	45.54 ± 0.79b	199.44 ± 13.30bc	10.62 ± 0.33a	5.44 ± 0.28ab	20.79 ± 1.89a	0.29 ± 0.04b

*Values are the mean ± SD, and those followed by same letter are not significantly different at p < 0.05.*

Figure 3B shows that the ethylene content in the control group increased rapidly on days 6-8, reached the highest point on day 10, and then declined slightly. However, the ethylene content of the treatment groups was significantly lower than that of the control group overall from days 6 to 10. The inhibition effect of treatment groups B and C was the best, in which the content declined by 41.21 and 38.65% on day 8 and 26.53 and 22.79% on day 10, respectively, indicating that ethanol treatment had an obvious effect on repression of the synthesis of ethylene, and that the effect varies with concentration.

Generally, the content of superoxide anions was increasing, and different concentrations had different effects (Figure 3C). At the later stage of storage, ethanol treatment can reduce the content of superoxide anions, and treatment group B has the best effect.

### Activities of Oxidases and Antioxidant Enzymes

**FIGURE 4:**
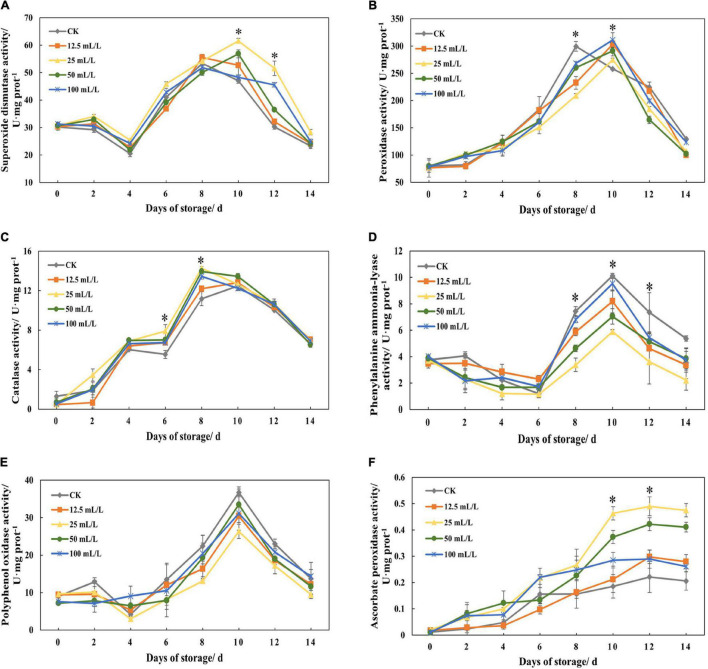
**(A)** Superoxide dismutase activity, **(B)** peroxidase activity, **(C)** catalase activity, **(D)** phenylalanine ammonia-lyase activity, **(E)** polyphenol oxidase activity, and **(F)** ascorbate peroxidase activity of papaya fruits treated with 0, 12.5, 25, 50, and 100 mL/L ethanol. Values are means ± SD. *Represents significant differences among the treatments (*p* < 0.05).

Figure 4A shows that the peak of SOD activity in the control group appeared on day 8, and that the peak in the experimental group was delayed until day 10. In the whole experiment, the activity of SOD in treatment group B was basically the highest, and the activities of the other treatment groups were also higher than that of the control group, which indicated that ethanol treatment could promote the activity of SOD.

Similarly, POD activity peak was delayed after ethanol treatment (Figure 4B). Overall, the POD activity of treatment B remained at a low level, while there was no marked difference between the other treatment groups and the control group, which suggested that suitable ethanol treatment concentration could effectively inhibit POD activity.

Figure 4C shows that CAT activity increased on days 2–4 and days 6–8, and decreased rapidly after the peak activity on day 8. In the first 8 days, the activity of treatment group B was the highest and then decreased, with a little difference from that of the other experimental groups, which showed that ethanol treatment mainly affected CAT activity at the first half of storage.

The activity of PAL decreased slightly on days 2–6, reached the highest when respiratory climacteric peaked, and then returned to the previous level (Figure 4D). During the latter half of the storage period, PAL activity was significantly reduced by ethanol treatment. Overall, treatment group B had the lowest activity and the best effect of inhibition.

Polyphenol oxidase activity increased rapidly on days 6-10 because of the maturity and senescence of the fruits and respiratory climacteric, while ethanol treatment could reduce the activity of enzymes to some extent compared with the control (Figure 4E). During the experiment, the activity of treatment group B was always low, followed by groups A and C, although the difference was not significant, indicating that the effect of ethanol on papaya fruit senescence was not mainly attributed to the inhibition of PPO activity.

It can be seen from Figure 4F that the activity of APX in treatment groups at the later stage of storage is higher than the activity of control group, and treatment groups B and C are 149.48% and 101.03% higher than control group according to Table 1. The increase in enzyme activity in these two treatment groups on days 8–10 is more obvious than that in the other treatment groups. It can be found that treatment with appropriate concentration of ethanol could significantly increase APX activity.

### Comprehensive Evaluation of Different Treatments Based on TOPSIS

For selecting the suitable concentration of postharvest papaya, we performed the Technique for Order of Preference by Similarity to Ideal Solution (TOPSIS) based on 15 physiological indicators storage day 10 (Table 2). The comprehensive score of treatment B was 0.99, significantly higher than that of the other groups, followed by treatment C (0.56) and control the group (0.53), while that of treatments A and D was lower than CK. It was obvious that treatment B (25 mL/L) was the suitable concentration of ethanol treatment in this study, and we suggested that high or low concentration of ethanol treatment was not conducive to the storage of papaya.

**TABLE 2:** Technique for Order of Preference by Similarity to Ideal Solution (TOPSIS) analysis of different treatments of based on 15 indicators on storage day 10.

Treatment	D_i_^+^	D_i_^–^	C	Rank
CK	32.15	35.65	0.53	3
A	56.35	20.85	0.27	4
B	0.84	61.13	0.99	1
C	28.75	36.00	0.56	2
D	48.40	14.11	0.23	5

### Principal Component Analysis (PCA) of Antioxidant System

A principal component analysis (PCA) was performed based on nine antioxidant indicators of papaya on storage day 10, with which exploring the main factors contribute to storage quality (Table 3). Three principal components were extracted with eigenvalues greater than 1 as standard, and variance contribution rates were as follows: 51.23, 14.74, and 12.52%, and cumulative contribution rate was 78.49%, and the three principal components could represent most of the filtered information. PC1 had the highest contribution rate, and the top three indicators were PAL, ethylene, and superoxide anions; the main influencing factors of PC2 were POD and MDA; PC3 was mainly affected by SOD, MDA, and APX.

**TABLE 3:** Principal component analysis of the antioxidant system on storage day 10.

	PC1	PC2	PC3
MDA	0.13	0.35	0.34
Ethylene	0.19	−0.17	0.11
Superoxide Anions	0.18	0.01	−0.09
SOD	−0.18	−0.04	0.38
POD	−0.04	0.42	−0.68
CAT	−0.09	−0.55	−0.29
PPO	0.13	−0.28	0.02
PAL	0.20	0.08	0.03
APX	−0.17	0.20	0.24
Eigenvalue	4.61	1.33	1.13
Variance contribution rate/%	51.23	14.74	12.52
Cumulative contribution rate/%	51.23	65.97	78.49

**FIGURE 5:**
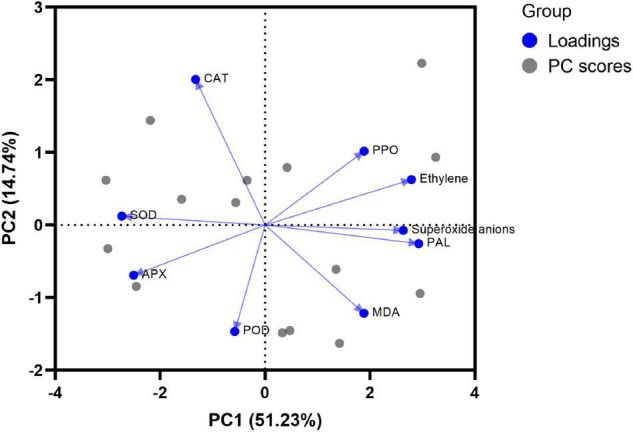
Biplot of principal component analysis of antioxidant system on storage day 10.

The biplot of the PCA is shown in Figure 5. The angles among PAL, ethylene, superoxide anions, MDA, and PPO were less than 90°, indicating that they were positively correlated. Also, they were on the positive half of PC1, which meant that these 5 indicators had a positive effect on PC1 and were not conducive to storage of postharvest papaya.

## Discussion

Climacteric fruits mature and age rapidly after harvest, and are accompanied by deterioration, water loss in the flesh, and entire quality decline. Ethanol, a green and safe food additive, could effectively delay fruit ripening and senescence, and improve the overall storage quality, which has been proved in a variety of horticultural crops and is a potent strategy for postharvest preservation. This study demonstrated that the storage quality of postharvest papaya was improved, and that the antioxidant system was promoted by ethanol treatment.

### Ethanol Treatment Improved the Appearance Quality of Postharvest Papaya

In this experiment, ethanol treatment could the delay color-changing of papaya fruits for several days, maintain firmness, and reduce fruit decay incidence and weight loss rate, which were beneficial for improving appearance quality. Capotorto et al. (2018) soaked fresh-cut fennel in 0.5% ethanol for 2 min and found that it effectively preserved the visual quality in air at 5°C for 6 days and significantly reduced the blackening of the chopped stem and shell surfaces. The results of Ji et al. (2021) on blueberries indicated that ethanol vapor treatment delayed the decline in firmness and destruction of the cell wall structure of fruits. We presumed that ethanol treatment could also delay papaya fruit firmness by suppressing the activities of pectic enzymes and cellulases like PG and β-glucosidase (β-Glu). However, the higher concentration of treatment (treatment D) affected the effect of storage. Vilar et al. (2019) concluded that 3, 5, and 7% (v/v) ethanol in combination with hydrothermal treatment shortened the shelf life of mangoes for ripening faster and were not recommended for export. We speculated that, in order to convert high concentrations of ethanol into acetaldehyde and ethyl acetate, pulp cells accelerated the anaerobic respiration rate and storage energy consumption, which affects the storage quality.

### Ethanol Treatment Suppressed Oxidative Stress and Biosynthesis of Ethylene

Oxidative stress is considered as the main cause of senescence and severe pathology (Sohal and Weindruch, 1996; Andreyev et al., 2005), while the content of MDA and superoxide anions in fruits can reflect the level of oxidative stress indirectly. Ethanol treatment reduced the content of MDA and superoxide anions, indicating that appropriate concentration of ethanol treatment may have a positive effect on alleviating oxidative stress and delaying fruit senescence. What is more, the results of Dong et al. (2016) on fresh-cut burdock and Lin et al. (2020) on bitter melon showed that ethanol treatment could maintain cell membrane integrity by reducing the content of MDA and superoxide anions, prolonging the storage time of the fruits.

Ethylene, a gaseous hormone, plays a significant role in regulating fruit ripening (Barry and Giovannoni, 2007), and reduction of ethylene biosynthesis will undoubtedly delay fruit ripening. It has been reported in several species that ethanol treatment could efficiently suppress the content of ethylene and postpone maturation. Exogenous ethanol treatment delayed the aging of postharvest broccoli by inhibiting the activity of ACS and ACO stimulated by ethylene and related genes expression simultaneously (Asoda et al., 2009). During the storage period of oriental sweet melon, ethanol treatment decreased the internal ethylene concentration and enhanced the production of ethyl ester (Liu et al., 2012). Thewes et al. (2021) treated two varieties of apples with ethanol and found that fruit ripening could be inhibited even in the presence of 150 ppm ethylene, and that 500 ppm ethanol was the most effective. In this experiment, the entire ethylene contents of the treatment groups were notably lower than that of the control group, especially on days 6–10. As a typical fruit of respiratory climacteric, decrease in the ethylene content of papaya naturally reduces the peak of respiratory climacteric, thus delaying fruit ripening and senescence.

### Effect of Ethanol Treatment on Oxidases and Antioxidant Enzymes

There are effective efficient enzymatic (SOD, CAT, APX, and so on) and non-enzymatic antioxidant defense systems in plants, which protect plant cells from oxidative damage by eliminating reactive oxygen species (Gill and Tuteja, 2010). SOD combined with CAT can transform superoxide free radical (O_2_^–^) into O_2_ and H_2_O; APX participates in the ascorbate-glutathione (AsA-GSH) cycle to remove H_2_O_2_, and these enzymes reduce the damage of ROS to the cell membrane (Apel and Hirt, 2004). This study concluded that proper concentration of ethanol treatment could promote the activities of SOD, CAT, and APX, and hasten the antioxidant system, which is finally manifested as delay of fruit ripening and senescence. These were similar to those found on banana treated with ethanol by de França et al. (2019), and further studies of Li et al. (2018) on fresh-cut strawberries found that exogenous ethanol treatment activated the related gene expression of SOD, CAT, and APX, thereby increasing activities of enzymes.

On the contrary, ethanol treatment inhibited the activities of POD, PPO, and PAL, which are the main enzymes inducing enzymatic browning in horticultural crops (Vamos-Vigyazo and Haard, 1981; Nicolas et al., 1994; Lopez-Serrano and Barcelo, 2002; Jhin and Hwang, 2015). Phenylalanine could be converted to ammonia and trans-cinnamic acid by PAL, while the latter could be ulteriorly converted to phenylpropanoid compounds, including monophenol and o-diphenol. These phenolic substances could be oxidized by PPO to o-diquinones and further oxidized to brown pigment or melanin, thus affecting the appearance and quality of horticultural crops. Exogenous ethanol treatment could restrain the activities of POD, PPO and PAL, reduce browning, and improve fruit quality after harvest, which were the same as the results of Hu et al. (2010) on fresh-cut eggplants and Dong et al. (2016) on fresh-cut burdocks. Similarly, ethanol treatment combined with acetic acid (Huang et al., 2020) and phytoncide (Kim et al., 2014) could notably inhibit the enzymatic browning of lettuce. In addition, PAL is one of the key enzymes regulating anthocyanin biosynthesis (Leyva et al., 1995; Boss et al., 1996), and decline in PAL activity slowed the process of color-changing in papaya.

### Major Contributors to Storage Quality and Fruit Ripening Based on PCA

The PCA reflected the contribution of each indicator to storage quality and fruit ripening. In this study, PC1 (51.23%) accounted for half contribution rate, of which three most important indicators were PAL, ethylene, and superoxide anions, followed by PC2 (14.74%) and PC3 (12.52%). It can be seen that the increase in PAL activity and ethylene and superoxide anion content were the main contributors for papaya fruit ripening and senescence after harvest, and that ethanol treatment suppressed PAL, ethylene, and superoxide anions, which delayed the maturation of the fruits.

## Conclusion

**FIGURE 6:**
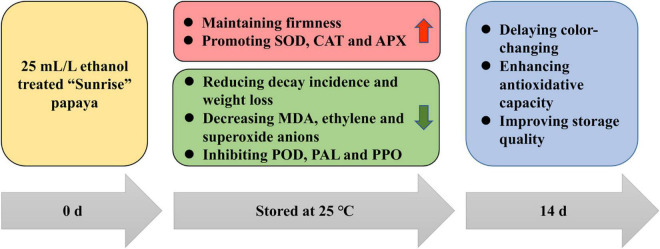
Putative action pattern of ethanol treatment on postharvest papaya.

The purpose of this study was to determine the effect of ethanol treatment on postharvest papaya and showed that ethanol soaking treatment could effectively delay color-changing, decrease decay incidence and water loss rate, and slow the decline of firmness to maintain the storage quality of papaya fruits. Moreover, ethanol treatment reduced the biosynthesis of MDA, ethylene, and superoxide anions, promoted the activities of SOD, CAT, and APX, and inhibited the activities of POD, PAL, and PPO, so as to hasten the antioxidant system and delay the maturation of postharvest papaya (Figure 6). In addition, that PCA showed that PAL, ethylene, and superoxide anions were the three most important indicators accounting for ripening and aging. In general, therefore, it seems that ethanol treatment has the potential of delaying the ripening and maintaining the storage quality of papaya fruits, and that treatment group B (25 mL/L ethanol) is the most suitable for papaya based on the TOPSIS analysis in this experiment. This study focused on physiological effects of ethanol treatment on papaya, and further studies should clarify the molecular regulation mechanism, in which RNA-seq could be performed for identifying main metabolism pathways and differentially expressed genes and choosing key genes for cloning and functional analysis.

## Data Availability Statement

The original contributions presented in this study are included in the article/supplementary material, further inquiries can be directed to the corresponding authors.

## Author Contributions

PC and ZL contributed to conception and designed the experiment. ZL, YM, and HL implemented the experiment. ZL processed and analyzed the data and wrote the first draft of the manuscript. FJ, PC, and HZ revised the manuscript and made modifications. PC supervised the whole study and provided guidance. PC and HZ acquired the funding. All authors have read and approved the manuscript.

## Conflict of Interest

The authors declare that the research was conducted in the absence of any commercial or financial relationships that could be construed as a potential conflict of interest.

## Publisher’s Note

All claims expressed in this article are solely those of the authors and do not necessarily represent those of their affiliated organizations, or those of the publisher, the editors and the reviewers. Any product that may be evaluated in this article, or claim that may be made by its manufacturer, is not guaranteed or endorsed by the publisher.
